# LiGaussOcc: Fully Self-Supervised 3D Semantic Occupancy Prediction from LiDAR via Gaussian Splatting

**DOI:** 10.3390/s25185889

**Published:** 2025-09-20

**Authors:** Zhiqiang Wei, Tao Huang, Fengdeng Zhang

**Affiliations:** 1 School of Optical-Electrical and Computer Engineering, University of Shanghai for Science and Technology, Shanghai 200093, China; 2 College of Science and Engineering, James Cook University, Cairns, QLD 4878, Australia

**Keywords:** 3D semantic occupancy, LiDAR perception, Gaussian rendering, self-supervision, autonomous driving, point cloud, voxelization

## Abstract

Accurate 3D semantic occupancy perception is critical for autonomous driving, enabling robust navigation in unstructured environments. While vision-based methods suffer from depth inaccuracies and lighting sensitivity, LiDAR-based approaches face challenges due to sparse data and dependence on expensive manual annotations. This work proposes LiGaussOcc, a novel self-supervised framework for dense LiDAR-based 3D semantic occupancy prediction. Our method first encodes LiDAR point clouds into voxel features and addresses sparsity via an Empty Voxel Inpainting (EVI) module, refined by an Adaptive Feature Fusion (AFF) module. During training, a Gaussian Primitive from Voxels (GPV) module generates parameters for 3D Gaussian Splatting, enabling efficient rendering of 2D depth and semantic maps. Supervision is achieved through photometric consistency across adjacent camera views and pseudo-labels from vision–language models, eliminating manual 3D annotations. Evaluated on the nuScenes-OpenOccupancy benchmark, LiGaussOcc achieved performance competitive with 30.4% Intersection over Union (IoU) and 14.1% mean Intersection over Union (mIoU). It reached 91.6% of the performance of the fully supervised LiDAR-based L-CONet, while completely eliminating the need for costly and labor-intensive manual 3D annotations. It excelled particularly in static environmental classes, such as drivable surfaces and man-made structures. This work presents a scalable, annotation-free solution for LiDAR-based 3D semantic occupancy perception.

## 1. Introduction

3D semantic occupancy perception, which provides a dense volumetric model of objects and drivable space, is a fundamental task in autonomous driving that directly enables robust motion planning and precise obstacle avoidance. In recent years, vision-based approaches have made remarkable progress in this domain, driven by innovations in architecture, efficiency, and supervision paradigms. Architectural advancements have explored a wide range of 3D representations. For example, BEVFormer introduced spatio-temporal transformers for Bird’s-Eye View (BEV) grids [1], while SurroundOcc demonstrated strong multi-camera performance using dense volumetric grids [2]. VoxFormer leveraged sparse voxel transformers for semantic scene completion [3], and others have refined 2D-to-3D lifting, such as decoupling height in occupancy queries [4] or applying depth-aware strategies for monocular prediction [5]. To improve computational efficiency, SparseOcc reduced redundancy through sparse representations [6], while SHTOcc addressed long-tail voxel distributions to enhance performance [7]. In terms of supervision, OccNeRF used neural radiance fields to guide occupancy learning [8], UniOcc combined geometric and semantic rendering [9], and SfmOcc introduced a novel structure-from-motion pipeline for pseudo ground truth generation [10]. This rapid progress has been supported by standardized evaluation on large-scale benchmarks such as OpenOccupancy [11]. Despite these advances, pure vision-based approaches suffer from fundamental limitations: inaccurate depth estimation leading to volumetric errors; high sensitivity to variations in ambient lighting; and lack of robustness in adverse weather conditions such as rain, snow, or fog.

Motivated by the inherent challenges of vision-centric occupancy perception, methodologies centered on LiDAR sensors have been developed. LiDAR provides accurate depth measurements and captures the 3D structure of the environment, exhibiting robust performance across varying light conditions and inclement weather. Recent studies have demonstrated the significant potential of LiDAR for predicting 3D semantic occupancy. For instance, PointOcc [12] introduced a cylindrical tri-perspective view specifically designed for LiDAR’s data distribution, achieving efficient prediction. Similarly, SSC-RS [13] proposed a novel framework that decouples the learning of semantic and geometric representations before fusing them in Bird’s-Eye View (BEV), a strategy effective for sparse point clouds. Further advancements include approaches like OccMamba [14], which employs state-space models to process large-scale 3D data efficiently, while LMSCNet [15] presents a lightweight architecture for real-time semantic completion. Others have explored novel representations, such as local deep implicit functions [16], to create continuous scene models that avoid voxelization artifacts. Despite these advances, significant challenges persist for LiDAR-based occupancy prediction. A primary limitation is the inherent sparsity of LiDAR points, which restricts the resolution of the predicted occupancy grid. Furthermore, training these models demands dense supervision, with the manual annotation of 3D ground truth being a notoriously expensive and time-consuming bottleneck. Consequently, many approaches rely on pseudo-labels generated from accumulated laser scans. However, this reliance on pseudo-labels from aggregated scans is inherently flawed. This strategy not only introduces systemic biases but also confines the model’s learning to the intrinsic sparsity of the sensor, as it is supervised by a denser version of its own input modality. Therefore, these limitations necessitate the development of novel frameworks capable of performing dense LiDAR-based occupancy prediction within a self-supervised paradigm, thereby removing the reliance on costly manual annotations.

To address these challenges, we propose LiGaussOcc, a novel self-supervised framework for LiDAR-based occupancy prediction that leverages differentiable Gaussian rendering to enable annotation-free training. We first voxelize multi-frame LiDAR point clouds through VoxelNet [17], and we employ the SECOND network [18] for encoding 3D voxel features. Compared with camera-based occupancy prediction, this process eliminates the depth errors and memory consumption associated with 2D-to-3D transformations [1,19]. Next, we introduce a novel module, Empty Voxel Inpainting (EVI), to optimize empty voxel features for addressing LiDAR sparsity and enhancing occupancy density. Furthermore, the Adaptive Feature Fusion (AFF) module is designed to enhance the dense voxel features of the EVI module with an adaptive fusion mechanism. During the training stage, we introduce the Gaussian Primitive from Voxels (GPV) module, which is tasked with generating Gaussian parameters (e.g., position μ, covariance σ, opacity α) for each voxel. Leveraging the prevalence of multi-view cameras on autonomous vehicles, these parameters are then used to render the 3D voxel representation onto 2D image planes, generating the dense depth and semantic maps required for our self-supervised objective. A naive approach to supervising the predicted occupancy would be to directly compute the loss between the rendered images and the corresponding camera images, similar to the 3D Gaussian Splatting [20] method. However, LiDAR point clouds lack the rich color information found in images. Another potential supervision method mentioned in GaussRender [21] is to utilize ground truth annotations from datasets. Although this approach can render 2D feature maps from different viewpoints, it either relies on expensive manual annotations [11] or uses depth information from aggregated multi-frame laser scans [2,22], which is clearly not suitable for training LiDAR-based occupancy prediction. To resolve these issues, we employ adjacent-view photometric loss as a supervision signal for self-supervised depth information with following GaussianOcc [23], and we obtain dense 2D semantic pseudo-labels through the vision language models (VLMs) as mentioned in OccNerf [8]. This dual-stream supervision strategy circumvents the need for error-prone 3D pseudo-labels, simultaneously mitigating the challenges of high annotation costs and inherent LiDAR sparsity.

The contributions of our paper are summarized as follows:

- A novel self-supervised and dense LiDAR-based framework for 3D semantic occupancy prediction is proposed. To the best of the authors’ knowledge, this is the first LiDAR-based architecture with Gaussian rendering for the 3D semantic occupancy prediction task. The proposed framework efficiently processes LiDAR data to generate dense 3D voxel representations that compensate for LiDAR modality limitations, thereby improving performance.
- For the inference pipeline, we propose two novel modules to facilitate a unified and fine-grained 3D semantic occupancy perception: an Empty Voxel Inpainting (EVI) module that densifies the initial sparse voxel features, and an Adaptive Feature Fusion (AFF) module that subsequently refines them via an adaptive fusion mechanism.
- To enable self-supervision, we introduce the Gaussian Primitive from Voxels (GPV) module, which serves as a bridge between the 3D and 2D domains. By predicting the parameters for 3D Gaussian Splatting, the GPV module facilitates the rendering of LiDAR voxels into dense 2D depth and semantic maps, thereby allowing for effective self-supervision using 2D annotations.
- Through extensive evaluation on the nuScenes-OpenOccupancy benchmark, we show that our self-supervised method achieved 30.4% Intersection over Union (IoU) and 14.1% mean IoU (mIoU). This performance reached 91.6% of the fully supervised LiDAR-based L-CONet, while entirely eliminating the need for costly manual 3D annotations. These results demonstrate the practicality and scalability of our approach for real-world 3D perception applications.

## 2. Related Work

This section reviews the relevant literature across the three interconnected research areas that formed the foundation of our work. We begin by discussing LiDAR-based 3D occupancy prediction methods, which address the core task of our framework. We then examine the recent development of 3D Gaussian Splatting, which serves as the primary representation mechanism in our approach. Finally, we review self-supervised learning strategies that leverage differentiable rendering, highlighting how these ideas intersect with our use of Gaussian-based supervision.

### 2.1. LiDAR 3D Occupancy Prediction

Significant advances in vision-based 3D occupancy prediction are marked by works such as MonoScene [24] for monocular 3D completion, TPVFormer [25] with its effective tri-perspective view representation, and the C-CONet [11] baseline, which lifts multiview image features into a unified 3D volume. However, these methods are fundamentally constrained by geometric inaccuracies from unreliable depth estimation and a lack of robustness in challenging lighting conditions or adverse weather conditions. This motivated the shift towards LiDAR-based methodologies; the sensor inherently provides accurate depth measurements and exhibits robust performance across these difficult environmental conditions. LiDAR-based occupancy prediction can be classified into two approaches based on how they represent and process the 3D scene [26]: (1) Projection-based methods, which improve efficiency by projecting sparse 3D data onto 2D pseudo-image representations, typically a Bird’s-Eye View (BEV) grid. These 2D feature maps are then processed through 2D convolutional neural networks (CNNs) or transformers before being lifted back to 3D [12,15,16]. These methods excel in balancing performance and efficiency, making them suitable for real-time applications. (2) The second approach involves 3D voxel-based methods; in contrast to projection, these methods operate directly on a 3D volumetric representation of the scene. They voxelize the point cloud and employ 3D CNNs to learn geometric and semantic features in 3D space [11,27,28]. This approach preserves 3D spatial relationships more faithfully, as demonstrated by powerful voxel-based methods including the fully supervised baseline L-CONet [11] and JS3C-Net [29], which learns contextual shape priors.

Despite significant progress, the existing methods suffer from critical limitations. Projection-based strategies typically trade 3D geometric fidelity for efficiency. Conversely, although voxel-based approaches better preserve 3D structure, they often lack explicit mechanisms to address the partial-to-complete estimation challenge that arises from the inherent sparsity of LiDAR scans. This reveals a critical research gap for a framework capable of inferring coherent object geometry from sparse observations, which directly motivated our design of the EVI and AFF modules.

### 2.2. Occupancy Prediction with 3D Gaussian Splatting

3D Gaussian Splatting (GS) [20] has recently emerged as a promising technique for 3D reconstruction, where learnable Gaussians serve as scene representations and improve training and rendering efficiency over voxel-based representations [8,30]. In contrast to conventional 3D GS that optimizes Gaussians independently for each scene, generalizable reconstruction methods [23] predict Gaussian parameters conditioned on image inputs in a feed-forward manner, enabling the learning of structural priors across multiple scenes. PixelSplat [31] pioneered generalizable 3D GS by sampling Gaussians from predicted probability distributions. Following this, efficient 3D Gaussian Splatting methods for autonomous driving were studied. GSRender [32] introduced a novel ray compensation module that intelligently samples rays and employs a sophisticated weak supervision scheme, which effectively mitigates duplicate occupancy predictions. Subsequently, GaussianFormer [33] and its successor GaussianFormer-2 [34] further advanced performance by integrating a sophisticated transformer-based architecture. GaussRender [21] develops a differentiable Gaussian rendering pipeline that enables direct optimization of Gaussian parameters and implicitly encodes occupancy features within 3D Gaussian representations; this approach applies supervision and improves performance for floating artifacts and poor surface localization. A crucial limitation of these methods, however, is their exclusive focus on vision modality. They are fundamentally designed for dense, textured camera inputs, and their underlying principles do not readily transfer to the sparse, texture-less nature of LiDAR data. Consequently, the application of 3D Gaussian Splatting to LiDAR-based occupancy prediction remains a significant, underexplored research area that our work aims to fill.

### 2.3. Self-Supervised 3D Occupancy Prediction with 3D Gaussian Splatting

While fully supervised training is effective for 3D occupancy prediction, it is often constrained by the need for large-scale, voxel-level manual annotations, which are prohibitively expensive to acquire. To circumvent this limitation, self-supervised learning has emerged as a promising direction, aiming to derive supervisory signals from readily available sensor data. A dominant early paradigm centered on volume rendering [30], where methods like OccNeRF [8] and selfocc [35] minimize photometric error between a warped source image and a target image. However, this approach can be computationally intensive and slow to converge, limiting its practical applicability. Recently, the advent of 3D Gaussian Splatting (3D-GS) [20] has introduced a new paradigm for high-quality, real-time rendering, which has been rapidly adopted for self-supervised occupancy prediction. Recent state-of-the-art methods include GaussOcc [23], which pioneers a fully self-supervised paradigm for 3D occupancy estimation by optimizing Gaussian Splatting kernels to minimize photometric reprojection errors, and GaussTR [36], which integrates Gaussian Splatting with vision transformers foundationally aligned with pre-trained visual encoders, enabling scalable self-supervised learning for cross-modal spatial understanding across diverse urban and indoor environments. Others, like GaussianPretrain [37], leverage 3D-GS to learn powerful general-purpose representations from large-scale unlabeled data. However, these approaches are inherently designed for camera inputs and rely heavily on rich color and texture information. Directly applying this principle to LiDAR-only setups is a non-trivial challenge due to the sparsity and lack of texture in point cloud data. To the best of the authors’ knowledge, how to effectively adapt the 3D-GS framework for robust, LiDAR-only self-supervision remains an open problem. This gap motivated the core design of our LiGaussOcc framework, which is specifically tailored to address the unique properties of LiDAR data.

## 3. Method

This section presents the architecture and methodology of the proposed LiGaussOcc framework. We begin by introducing the core rendering principle, 3D Gaussian Splatting, which underpins our self-supervised learning paradigm. We then detail the components of the LiDAR-only inference pipeline, including the novel Empty Voxel Inpainting (EVI) and Adaptive Feature Fusion (AFF) modules, both designed to address the inherent sparsity of LiDAR data. Next, we describe the self-supervised training mechanism, in which the Gaussian Primitive from Voxels (GPV) module transforms voxel features into differentiable Gaussian primitives. These primitives are rendered into 2D depth and semantic maps, which are supervised using photometric consistency loss (Ldep) and semantic pseudo-labels provided by vision foundation models (Lsem), respectively.

### 3.1. Preliminary for 3D Gaussian Splatting

In this study, 3D Gaussian Splatting [20] is employed as a foundational method for achieving fully self-supervised 3D semantic occupancy prediction. This innovative technique represents and renders 3D scenes using a collection of point primitives, with each primitive modeled by the following distribution:

(1) $$G\left(x\right)=e^{-\frac{1}{2}{\left(x-\mu \right)}^{T}\Sigma^{-1}\left(x-\mu \right)}$$

where $\mu \in R^{3}$ denotes the unique mean position of a primitive, and where Σ represents the 3D covariance matrix. For differentiable optimization, Σ is decomposed into a learnable scaling matrix $S\in R_{+}^{3}$ and a rotation matrix $R\in R^{4}$:

(2) $$\Sigma_{3D}=RSS^{T}R^{T}$$

Subsequently, the 3D Gaussians are projected onto a 2D image plane via a view transformation *W* and the Jacobian *J* of the projective approximation, thereby yielding the corresponding 2D covariance:

(3) $$\Sigma_{2D}=JW\Sigma_{3D}W^{T}J^{T}$$

Following the 2D projection, an alpha-blend rendering technique is adopted to determine the final pixel color. The accumulated color $C_{\mathrm{color}}$ is formulated as a weighted sum:

(4) $$C_{\mathrm{color}}=\sum_{i\in N}c_{i}\alpha_{i}\prod_{j=1}^{i-1}\left(1-\alpha_{j}\right)$$

where $c_{i}$ denotes spherical harmonics (SH) representing the color associated with each Gaussian, and where $\alpha_{i}$ is the product of learned opacity and the 2D Gaussian defined by covariance Σ^’^.

In summary, each Gaussian primitive is parameterized by these essential elements:

- 3D position: $\mu \in R^{3}$;
- Color defined by SH coefficients with *k* dimentions: $c\in R^{k}$;
- Rotation represented by a quaternion: $r\in R^{4}$;
- Scale: $s\in R_{+}^{3}$;
- Opacity: $\alpha \in \left[0,1\right]$.

### 3.2. Architecture

**Figure 1:**
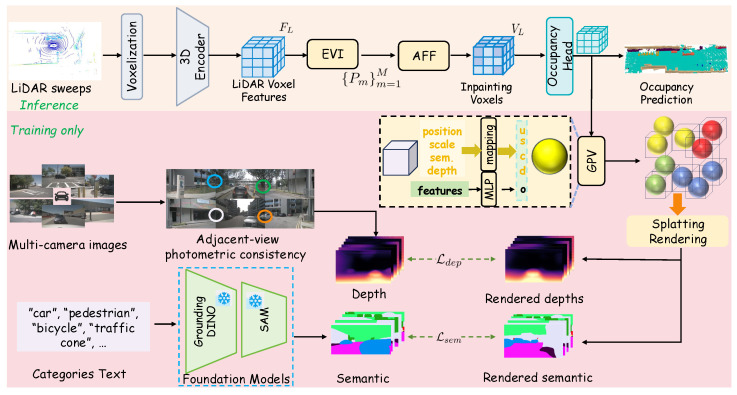
The overall architecture of our LiGaussOcc framework, showing the LiDAR-only inference pipeline (**top**) and the self-supervised training pipeline (**bottom**) that uses multi-view images for supervision.

The proposed LiGaussOcc is a novel, self-supervised framework for LiDAR-based occupancy prediction that employs differentiable Gaussian rendering technology to generate dense representations. The overall architecture is illustrated in Figure 1 and consists of two primary stages. The top section depicts the inference pipeline: multi-sweep LiDAR point clouds are voxelized and passed through the proposed EVI and AFF modules to generate a dense representation, from which the final 3D occupancy grid is predicted. The bottom section outlines the self-supervised training stage: voxel features are processed by the GPV module to generate parameters for 3D Gaussian Splatting. These parameters are rendered into 2D depth and semantic maps, which are supervised using photometric consistency ($L_{dep}$) and pseudo-labels derived from foundation models ($L_{sem}$), respectively.

For inference, unstructured LiDAR point clouds are initially encoded into sparse 3D voxel features $F_{L}\in R^{C\times D\times H\times W}$ through voxelization [17] and the Second network [18], where *D*, *H*, *W* denotes the volumetric dimensions of the scene, and where *C* represents the feature dimension of each LiDAR voxel. To address the inherent sparsity of LiDAR voxels, an Empty Voxel Inpainting (EVI) module is introduced to regularize empty voxels, thereby generating dense LiDAR occupancy; then, an Adaptive Feature Fusion (AFF) module is proposed to refine the final LiDAR voxel features.

To facilitate dense supervision for the generated dense LiDAR occupancy during the training phase, a novel self-supervision mechanism is designed within the 2D dense domain, leveraging 3D Gaussian Splatting [20]. Specifically, a Gaussian Attributes Generation from Voxel (GAGV) module is first designed to extract the essential attributes for 3D Gaussian Splatting from the voxel features. Subsequently, splatting rendering is performed to project these voxels from the occupancy fields onto 2D image planes, yielding both depth maps $\hat{D}$ and semantic maps $\hat{S}$. Concurrently, following [8,23], multi-view RGB images enable supervision through adjacent-view photometric loss D for $\hat{D}$, while pre-trained foundation models generate semantic pseudo-labels S to supervise $\hat{S}$.

The decoupled architecture in Figure 1 represents an optimal design, strategically engineered to resolve the dual challenges of LiDAR sparsity and the need for training without manual annotations. The inference pipeline (top) is dedicated to the spatial domain, progressively densifying the sparse LiDAR input into a coherent voxel representation using the EVI and AFF modules. The training pipeline (bottom), in contrast, operates as a supervision bridge. It projects the 3D voxel features into the 2D image domain via differentiable Gaussian rendering, a critical step that enables a self-supervised paradigm. This allows the network, which starts with sparse 3D data, to be guided by powerful, dense supervision signals derived from cameras. This strategic separation is crucial: it allows the inference path to focus solely on the geometric task of densification, while the complex machinery of cross-domain supervision is handled entirely offline during training.

### 3.3. EVI Module

**Figure 2:**
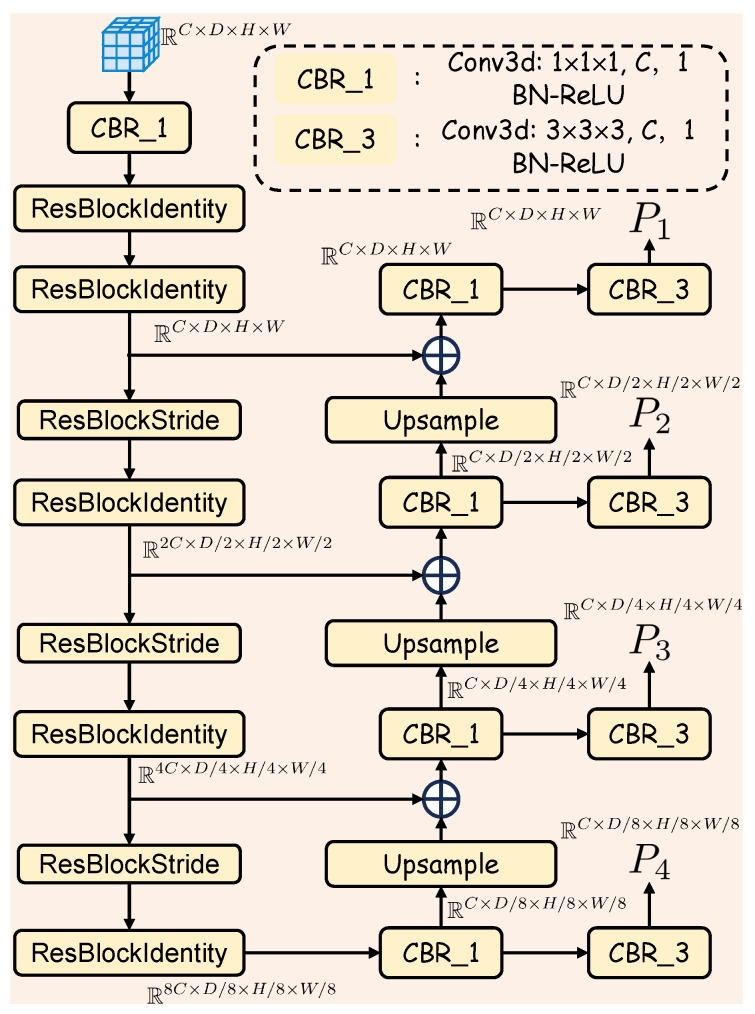
Illustration of EVI module. Voxel feature inpainting is achieved using a combination of 3D ResNet blocks and 3D deconvolution layers.

**Figure 3:**
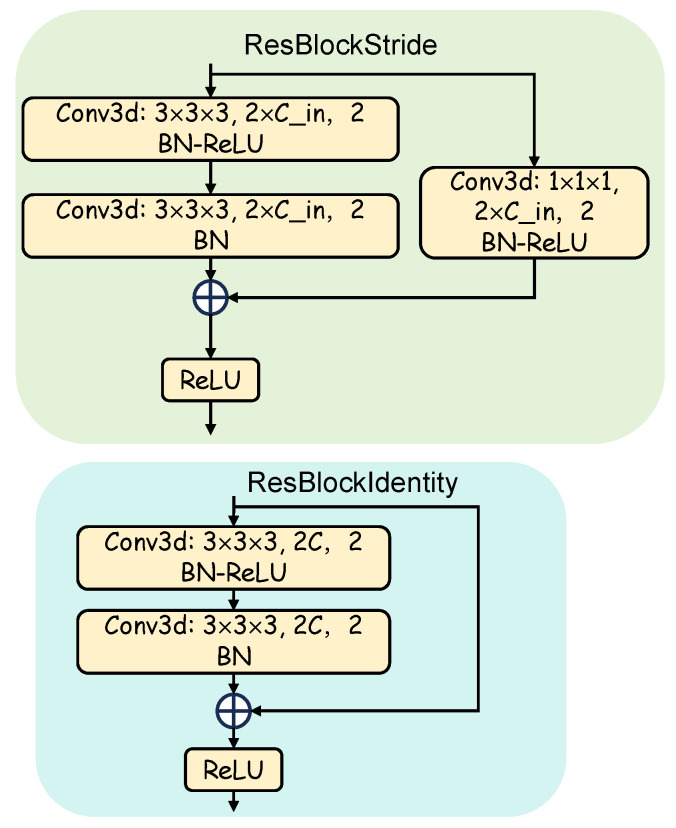
Detailed illustration of the ResBlockStride and ResBlockIdentity blocks, which are the core components of the EVI module.

To overcome the inherent sparsity of LiDAR data for dense occupancy prediction, we introduce the Empty Voxel Inpainting (EVI) module, an encoder–decoder architecture inspired by principles from image inpainting [38,39]. As illustrated in Figure 2, with the detailed components shown in Figure 3, the module transforms the sparse voxel grid into a dense feature representation via a two-stage process. First, a 3D ResNet-based [40,41] encoder is employed to extract multi-scale 3D voxel features ${\left\{V_{L}\in R^{C_{m}\times D_{m}\times H_{m}\times W_{m}}\right\}}_{m=1}^{M}$, where *m* denotes the scale level, and where $D_{m}$, $H_{m}$, $W_{m}$, and $C_{m}$ represent the depth, height, width, and channel dimensions of the *m*-th scale feature map, respectively. This encoder progressively downsamples the voxel features, enlarging the receptive field at each stage. This enables the network to learn high-level contextual information from broader spatial regions, allowing it to infer the underlying structure of the scene even from sparsely populated points. Subsequently, a decoder path, structured similarly to a 3D Feature Pyramid Network (FPN) [42,43], upsamples the encoded features $Y_{j}$ from the *j*-th level, using 3D deconvolution layers. These upsampled features are fused with the corresponding encoder features $Y_{j-1}$ via skip connections. This fusion is critical, as it enables the decoder to leverage both global contextual information and fine-grained spatial detail. The combined features are then further refined using 3D convolutions. The final output consists of densified multi-scale feature maps ${\left\{P_{m}\in R^{C_{m}\times D_{m}\times H_{m}\times W_{m}}\right\}}_{m=1}^{M}$, where *M* is set to 4 in our LiGaussOcc.

The EVI module employs an encoder–decoder architecture with skip connections, a topology proven effective in image inpainting for its ability to synthesize plausible content. This design is optimal for handling LiDAR sparsity because it fuses two critical streams of information. The encoder path captures high-level semantic context across progressively larger receptive fields, while the skip connections preserve and reintroduce fine-grained spatial details to the decoder. This fusion of contextual and spatial information enables the module to plausibly reconstruct features within unobserved empty voxels, ultimately yielding a coherent and dense scene representation.

### 3.4. AFF

**Figure 4:**
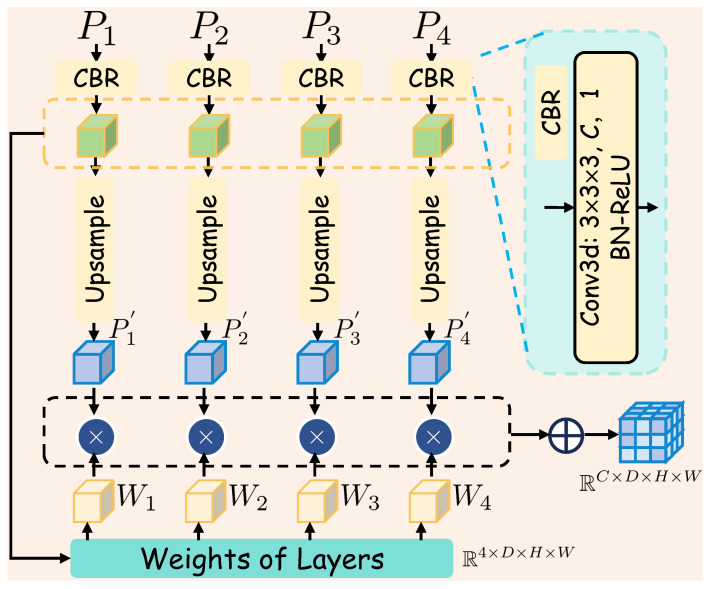
Illustration of Adaptive Feature Fusion. To enhance the final 3D voxel features, we implement an adaptive fusion mechanism, where features from multiple scales are dynamically weighted and subsequently integrated.

As illustrated in Figure 4, the multi-scale LiDAR voxel features from each level, $P_{m}$, are fused with learned weights through an adaptive fusion mechanism. This adaptive fusion dynamically combines information from different scales to synthesize the final dense occupancy representations $V_{L}$, effectively inpainting the empty voxels by leveraging diverse contextual and detailed information across the feature hierarchy. The fusion mechanism is formulated as follows:

(5) $$V_{L}=\sum_{m=1}^{M}P_{m}^{\prime }\times W_{m},$$

where

(6) $$P_{m}^{\prime }=\gamma \left(P_{m}\right),$$

(7) $$W_{m}=\sigma \left(\mathrm{Conv}\left(P_{m}\right)\right).$$

Here, $\gamma \left(\cdot \right)$ represents the upsample operation, and Conv(·) denotes the weight encoding network, which primarily consists of a 3D convolutional network and normalization layers. The weight $W_{m}$ is computed via a sigmoid function and is used to balance the contribution from different scale voxel features, with values ranging from 0 to 1.

The superiority of the proposed adaptive fusion mechanism over simpler alternatives, such as summation or concatenation, was quantitatively validated by our ablation study. The results confirm that by using learnable weights to dynamically emphasize features from the most informative scales, our network produces a more robust and refined final representation capable of handling the diverse structures in driving environments.

### 3.5. GPV Module

To facilitate the conversion of voxel-based occupancy representations into differentiable Gaussian primitives, the Gaussian Primitive from Voxels (GPV) module is designed with a focus on simplified Gaussian parameterization, as depicted in Figure 1. This approach aims to prevent unstable configurations during the learning process and to streamline optimization. The requisite attributes for each Gaussian primitive are extracted from its associated voxel at position μ=(x,y,z). These attributes include the following:

- the 3D position μ: directly inherited from the voxel’s grid coordinates μ, eliminating learnable position offsets;
- scale *S*: defined as a diagonal matrix Diag(s), where *s* is a scalar factor determined by the voxel’s dimensions;
- color logits *c*: directly derived from the model’s final occupancy semantic prediction for the corresponding voxel, preserving class probability distributions;
- opacity *o*: a learned parameter with implementing MLP for voxel features;
- rotation matrix *R*: fixed with identity matrix *I*, reducing the covariance matrix to $\Sigma_{3D}=S^{2}$, and ensuring spherical symmetry.

### 3.6. Rendering with Gaussian Splatting

To enhance the efficiency of the rendering pipeline, a 3D Gaussian Splatting approach is employed to project occupancy voxels onto the 2D image domain. This process involves transforming the 3D covariance matrix $\Sigma_{3D}$ into the image plane with a given viewing transformation *W*, and the projected 2D covariance is defined as

(8) $$\Sigma_{2D}=J\cdot W\cdot \Sigma_{3D}\cdot W^{T}\cdot J^{T},$$

where *J* represents the Jacobian matrix, the affine approximation of the projective transformation [20]. The resulting 2D covariance represents the spatial distribution and shape of the Gaussian splats, directly governing per-pixel opacity $\alpha_{i}$ and transmittance $T_{i}$ computations during rasterization. For pixel *p*, the aggregated semantic color ${\hat{S}}_{p}\in {\left[0,1\right]}^{C}$, where C denotes the number of semantic classes, and the rendered depth ${\hat{D}}_{p}\in R^{+}$ are derived by summing contributions from all the overlapping Gaussians:

(9) $${\hat{S}}_{p}=\sum_{i=1}^{N}T_{i},\alpha_{i},c_{i},$$

(10) $${\hat{D}}_{p}=\sum_{i=1}^{N}T_{i},\alpha_{i},d_{i},$$

where *N* indicates the total number of overlapping Gaussians, $\alpha_{i}=1-exp\left(-\sigma_{i}\delta_{i}\right)$ represents the opacity via density $\sigma_{i}$ and ray interval $\delta_{i}$, $T_{i}=\prod_{j=1}^{i-1}\left(1-\alpha_{j}\right)$ denotes cumulative transmittance to address occlusion, and $d_{i}$ denotes the distance of the *i*-th Gaussian to the camera. Thus, the rendered semantic images $I_{\mathrm{sem}}$ and depth images $I_{\mathrm{dep}}$ can be represented as follows:

(11) $${\hat{I}}_{\mathrm{sem}}=\left\{{\hat{S}}_{p},p\in P^{*}\right\},$$

(12) $${\hat{I}}_{\mathrm{dep}}=\left\{{\hat{D}}_{p},p\in P^{*}\right\},$$

where $P^{*}$ represents the associated pixel set of each camera. This differentiable rendering mechanism enables direct projection of 3D semantic occupancy fields into geometrically consistent dense 2D representations; the 3D spatial information is effectively translated into 2D views, facilitating pixel-level supervision for LiDAR-based occupancy prediction.

### 3.7. Adjacent-View Photometric Consistency for Depth Label

To enforce geometrically consistent depth learning across multi-sensor platforms, our approach, by following [23], establishes adjacent-view photometric consistency constraints leveraging reprojection geometry. For each target view *i* and adjacent view *j*, an overlap mask is computed to isolate mutually visible regions while preserving scene completeness through unprojection. The pixel $p_{t}^{i}$ in view *i* is reprojected to coordinate $p_{t}^{i\to j}$ in view *j* via

(13) $$p_{t}^{i\to j}=K^{j}{\left(T^{j}\right)}^{-1}T^{i}D_{t}^{i}{\left(K^{i}\right)}^{-1}p_{t}^{i},$$

where $K^{i}$, $K^{j}$ denote intrinsic matrices, $T^{i}$, $T^{j}$ represent camera-to-world extrinsics, and $D_{i}^{t}$ is the predicted depth map of *i*th camera. Differentiable splatting renders the warped image ${\tilde{I}}_{\mathrm{dep}}$, which is similar to Equation (10). Accurate depth prediction ensures that ${\tilde{I}}_{\mathrm{dep}}$ photometrically aligns with the raw image *I*, providing depth-aware supervision.

### 3.8. Foundation Models for Semantic Label

**Figure 5:**
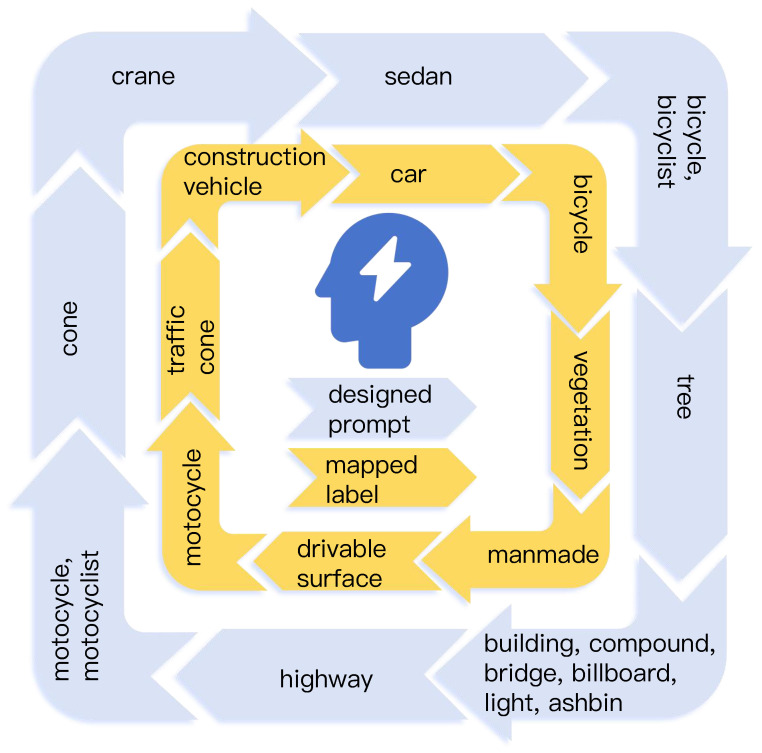
Illustration of prompt design. A specific text prompt is carefully designed for each semantic category to ensure accurate correspondence with the original class definitions of the dataset.

By following [8], we leverage a pre-trained open-vocabulary model Grounded-SAM to generate 2D semantic segmentation labels. The pre-trained open-vocabulary model enables us to obtain 2D labels that closely match the semantics of the given category names. The detailed promote design is shown in Figure 5. The design of these text prompts is crucial for maximizing the model’s performance. An abstract class name—for example, the ‘manmade’ category—can be ambiguous to the model. We found that providing a list of concrete, descriptive synonyms including ‘building’, ‘compound’, and ‘bridge’ elicits far more accurate and reliable segmentation masks. This process significantly improves the quality of the pseudo-labels that underpin our self-supervised training.

An uncertain label is given if the corresponding pixel does not belong to any category. Each pixel has ${\left\{g_{k}\right\}}_{k=1}^{K}$, where *K* is the number of categories; the per-pixel label $S_{p}$ is given by

(14) $$S_{p}=\chi \left(arg\max_{k}g_{k}\right),$$

where χ is a function that maps the index of $g_{k}$ to the category label according to the phrase. By following Equation (9), ${\tilde{S}}_{\mathrm{sem}}$ denotes the pseudo-label.

### 3.9. Loss Function

The depth loss $L_{\mathrm{dep}}$ and the semantic loss $L_{\mathrm{sem}}$ are shown as below:

(15) $$L_{\mathrm{dep}}=\frac{\beta }{2}\left(1-\mathrm{SSIM}\left({\tilde{I}}_{\mathrm{dep}},{\hat{I}}_{\mathrm{dep}}\right)\right)+\left(1-\beta \right){∥{\tilde{I}}_{\mathrm{dep}},{\hat{I}}_{\mathrm{dep}}∥}_{1},$$

(16) $$L_{\mathrm{sem}}=L_{\mathrm{ce}}\left({\hat{S}}_{\mathrm{sem}},{\tilde{S}}_{\mathrm{sem}}\right),$$

where β is set to 0.85 for balancing the two loss terms of $L_{\mathrm{dep}}$, and where $L_{\mathrm{ce}}$ represents the cross-entropy loss function. Our overall loss function $L_{\mathrm{total}}$ is expressed as

(17) $$L_{\mathrm{total}}=L_{\mathrm{dep}}+L_{\mathrm{sem}}.$$

## 4. Results

This section presents the experimental validation of our proposed LiGaussOcc framework. We first describe the experimental setup, including the dataset, evaluation metrics, and implementation details. We then present our main quantitative results, comparing our method against state-of-the-art fully supervised approaches. Finally, we provide a series of ablation studies to analyze the individual contributions of our key modules and supervision strategies.

### 4.1. Experimental Setup

#### 4.1.1. Dataset and Metrics

We evaluated our proposed LiGaussOcc framework on the nuScenes-OpenOccupancy dataset [11,44], a large-scale benchmark for 3D occupancy prediction. The dataset includes 1000 driving scenes, split into 700 for training, 150 for validation, and 150 for testing. Ground truth was provided as dense voxel grids of size (512,512,40) with 0.2 m resolution, covering 16 semantic categories and a free-space class. Following the standard evaluation protocol [11], we adopted Intersection over Union (IoU) to assess geometric completion and mean IoU (mIoU) for semantic prediction. These metrics are defined as

(18) $$IoU=\frac{TP}{TP+FP+FN},$$

(19) $$mIoU=\frac{1}{S}\sum_{i=1}^{S}\frac{TP_{i}}{TP_{i}+FP_{i}+FN_{i}},$$

where TP, FP, and FN denote the number of true positives, false positives, and false negatives, and where *S* is the number of semantic classes.

#### 4.1.2. Implementation Details

Our model processed a sequence of 10 accumulated LiDAR sweeps, which were voxelized and then passed through a voxel-based encoder for feature extraction [17,18]. The EVI module, designed to densify the sparse LiDAR features, was constructed using a 3D ResNet encoder and a 3D FPN decoder [40,41,42,43], enabling the inpainting of empty voxels based on contextual information. The AFF module employed a lightweight weight-generation network comprising a 1×1×1 3D convolution followed by normalization to adaptively fuse multi-scale features. Gaussian primitives were initialized with a scale of 0.1. For training supervision, we adopted 2D pseudo-labels generated using the semantic mapping approach from OccNeRF [8] and depth estimation from GaussianOcc [23].

The model was implemented using the MMDetection3D framework [45]. Training was performed for 20 epochs using the AdamW optimizer [46], which included a weight decay of $1\times 10^{-3}$ to regularize the model and reduce overfitting. An initial learning rate of $2\times 10^{-4}$ was used, with a batch size of 8 distributed across eight NVIDIA L20 GPUs. In addition, standard LiDAR data augmentation techniques from the MMDetection3D pipeline were employed to further mitigate overfitting.

### 4.2. Main Results

A comprehensive evaluation was conducted to benchmark our proposed self-supervised framework, LiGaussOcc, against state-of-the-art methods, with the results detailed in Table 1. Our method achieved a highly competitive IoU of 30.4 and mIoU of 14.1. It is critical to note that these results were obtained without relying on any manually annotated 3D ground truth for supervision, which fundamentally distinguishes our approach from traditional fully supervised methods and marks a significant contribution to the field. The significance of our method’s performance is further highlighted by its consistent and substantial gains across several key semantic classes critical for autonomous driving safety. As shown in Table 1, our method achieved state-of-the-art results among even some fully supervised methods in static environmental classes, including drivable surface (35.4% IoU), sidewalk (22.6% IoU), man-made structures (19.6% IoU), and vegetation (23.8% IoU). This consistent outperformance across multiple diverse categories, rather than just an aggregate mIoU improvement, provides strong evidence for the robustness and statistically significant superiority of our proposed framework. In addition, these improvements are also critical for autonomous driving safety, as a precise and coherent understanding of the static scene is fundamental for trajectory prediction and motion planning.

The effectiveness of our framework was further validated through direct comparisons with leading methods across different modalities. When compared with leading LiDAR-only methods, the result was particularly remarkable. Our self-supervised LiGaussOcc achieved 91.6% of the performance of the fully supervised state-of-the-art L-CONet. Closing the performance gap to this extent, without any 3D labels, demonstrates the immense potential and effectiveness of our proposed annotation-free training paradigm for LiDAR occupancy prediction. Against representative camera-only approaches, LiGaussOcc established a clear advantage in geometric and semantic understanding. For instance, it surpassed C-CONet by a significant 13.7% in mIoU. This highlights the inherent benefits of the LiDAR modality for 3D perception and validates our model’s ability to effectively process these sparse inputs into a dense, accurate representation. The ability of our self-supervised framework to achieve results nearly on a par with fully supervised LiDAR methods, while significantly outperforming camera-based ones and demonstrating proficiency in static scene understanding, highlights the power of our annotation-free paradigm. This represents a valuable and practical step towards building scalable, data-efficient, and robust 3D perception systems for autonomous driving.

**Figure 6:**
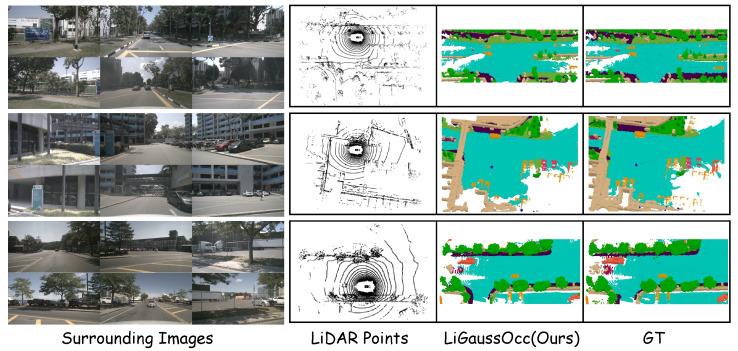
Visualization of qualitative results on the nuScenes-OpenOccupancy dataset [11,44]. The leftmost column shows the surround-view images, while the next three columns present original LiDAR points, semantic occupancy predictions from LiGaussOcc (ours), and the ground truth annotation, respectively. Please zoom in for finer details.

The qualitative results of LiGaussOcc are presented in Figure 6. The visualizations illustrate that LiGaussOcc yielded 3D occupancy predictions with fewer false positives. Moreover, our method demonstrated robust performance in perceiving static scene elements, accurately predicting classes like drivable surface, man-made structures, vegetation, and sidewalk in close alignment with the ground truth.

### 4.3. Ablation Study

**Ablation of Core Components.** A comprehensive ablation study was conducted to validate the individual contributions of our core components, the EVI and AFF modules, with the quantitative results presented in Table 2. Our analysis started from the full LiGaussOcc model, and it describes the performance degradation as each component was individually ablated. Firstly, to evaluate the impact of the EVI module, we replaced it with a standard downsampling block composed of four 3D convolutions. This change caused a substantial performance drop, with the mIoU decreasing by 11.3%. This result underscores the critical role of our EVI module in effectively processing sparse point cloud features to generate a high-quality scene representation. Secondly, we examined the effectiveness of the AFF module. By removing it and exclusively using the $P_{1}$ feature from the EVI module for subsequent processing (as depicted in Figure 2), the mIoU decreased by 6.4%. This degradation confirms that the feature fusion performed by the AFF module is essential for refining the occupancy prediction. These results demonstrate that each proposed module provides a distinct and critical contribution. The fact that the complete model achieved the highest score highlights a clear synergistic effect, validating our core components design.

**Ablation of Supervision Method.** Ablation studies were conducted to evaluate the impact of different supervision methods on the overall model performance, including ground truth supervision, volume rendering supervision, and our splatting rendering supervision, as presented in Table 3. First;y, we established a fully supervised upper bound by training our network architecture directly with the ground truth 3D labels from the OpenOccupancy dataset [11]. This fully supervised setup achieved a strong mIoU of 15.8. In contrast, our proposed method, which leverages 3D Gaussian Splatting for self-supervision, yielded a competitive mIoU of 14.1. This result is highly favorable compared to the alternative supervision strategies. It represents a 2.9% relative improvement over the baseline that uses volume rendering, a technique similar to that in OccNerf [8], confirming the superiority of our chosen rendering technique for generating high-quality supervisory signals. Most critically, our self-supervised result achieved 89.2% of the performance of the fully supervised upper bound. This demonstrates that our proposed framework effectively captures a substantial portion of the performance achievable with perfect 3D labels, marking a significant step towards annotation-free and data-efficient 3D perception.

**Ablation of AFF Module.** An ablation study was conducted to validate the fusion strategy of our proposed Adaptive Feature Fusion (AFF) module, with the results presented in Table 4. To evaluate its effectiveness, AFF was compared against two common fusion baselines adapted from methods in multi-modal fusion [47]. The baselines were as follows: (1) summation-based fusion, where multi-scale voxel features are upsampled and then combined via element-wise addition before being processed by an MLP; and (2) concatenation-based fusion, where the upsampled features are concatenated along the channel dimension and, subsequently, fed into an MLP. The experimental results clearly demonstrate the superiority of our proposed AFF module. It achieved the best performance, with 30.4 in SC IoU and 14.1 in mIoU. Specifically, AFF outperformed the stronger summation-based baseline by 3.7% in mIoU, and it surpassed the concatenation-based method by a more significant 7.6%. This validates that the adaptive weighting mechanism within our AFF module provides a more effective and robust feature fusion than conventional, non-adaptive strategies.

## 5. Discussion

Our experiments validated that LiGaussOcc effectively addresses the key challenges of LiDAR sparsity and annotation dependency in 3D occupancy prediction. By integrating a novel self-supervised paradigm with Gaussian rendering, our framework achieved a competitive 30.4% IoU and 14.1% mIoU on the nuScenes-OpenOccupancy benchmark. This performance was notably close to fully supervised methods, achieving 91.6% of L-CONet’s mIoU and, thereby, highlighting the viability of our annotation-free approach as a scalable alternative to methods reliant on expensive manual 3D labels.

Several key design choices underpinned the strong performance of our framework. The effectiveness of the inference architecture was validated through ablation studies (Table 2), which showed that removing the EVI module resulted in an 11.3% drop in mIoU, while ablating the AFF module led to a 6.4% degradation. These results demonstrate the critical role of these modules in densifying and refining sparse features. Additionally, Gaussian rendering supervision outperformed the volume rendering alternatives by 2.9% mIoU, highlighting its efficacy in generating dense 2D supervisory signals from sparse inputs. This advantage is reflected in the strong per-class results for static scene understanding, including a state-of-the-art 35.4% IoU for drivable surfaces.

When contextualized against other state-of-the-art methods, the merits of our approach become more evident. Our method achieved 14.1% mIoU, outperforming the camera-based C-CONet, which reached 12.4% mIoU, thereby confirming the superior geometric fidelity of the LiDAR modality. Furthermore, although our supervision strategy draws inspiration from vision-based methods such as OccNeRF, our framework is specifically designed to handle the sparsity challenges of LiDAR data, a constraint not encountered by camera-based approaches that operate on dense image features.

Despite these strengths, we acknowledge several limitations of the current framework. Firstly, performance remains limited for small and distant objects (e.g., 3.3% IoU for bicycles), primarily due to the inherent sparsity of LiDAR signals. Secondly, a key limitation lies in the reliance of our training paradigm on camera data, which can be unreliable under adverse conditions. Nonetheless, this limitation is mitigated by the scale and diversity of the nuScenes dataset, enabling the model to learn generalizable features despite occasional noisy frames. Camera-based signals serve as an indispensable and scalable teacher for learning a mapping from LiDAR structures to dense 3D semantics. This task is otherwise infeasible using sparse LiDAR data alone in a self-supervised setting. Finally, a critical consideration for real-world deployment is the computational cost of the 3D CNN backbone during inference, which can hinder real-time performance. While the Gaussian rendering-based training process is computationally intensive, it is conducted offline and does not affect deployment efficiency.

These limitations suggest clear and promising directions for future research. One key direction is to enhance perception robustness in adverse weather and for small objects by incorporating 4D radar. We plan to explore early-fusion strategies, where a dedicated network branch processes the radar’s velocity and weather-resilient data. These features could then be fused with LiDAR data at the voxel level using a cross-attention mechanism, allowing the model to dynamically weigh sensor inputs based on environmental conditions. Furthermore, addressing the computational bottleneck is critical for real-world deployment. Future work will, therefore, focus on model compression, specifically using knowledge distillation to transfer the representation from our EVI module into a lightweight student network to bridge the gap to real-time on-vehicle deployment.

## 6. Conclusions

In this paper, we introduced LiGaussOcc, a novel self-supervised framework designed to address the significant challenges of sparsity and 3D annotation dependency in LiDAR-based semantic occupancy prediction. Our core contribution is a new paradigm that utilizes 3D Gaussian Splatting to differentiably render LiDAR voxel features onto 2D image planes, enabling effective self-supervision from multi-view cameras without any 3D ground truth labels. The framework is further enhanced by specialized modules, EVI and AFF, designed to handle the inherent sparsity of LiDAR data during inference. Our extensive experiments conducted on the nuScenes-OpenOccupancy benchmark validated the effectiveness of our approach. LiGaussOcc achieved a highly competitive mIoU of 14.1% while also attaining state-of-the-art performance on critical static environmental classes such as drivable surfaces and vegetation. This performance was remarkably close to that of fully supervised state-of-the-art methods, demonstrating the viability of our annotation-free paradigm.

Despite the promising results achieved by LiGaussOcc, several limitations remain and define a clear roadmap for future research. The reliance on camera data for training introduces potential vulnerabilities under adverse environmental conditions, and the 3D CNN backbone imposes a significant computational burden that may hinder real-time deployment. Future work will address these challenges by exploring more robust supervision strategies, such as 4D radar integration and improving runtime efficiency through model compression techniques, including quantization and knowledge distillation. In conclusion, by successfully adapting differentiable rendering for LiDAR-based self-supervision, LiGaussOcc opens up a promising direction for developing scalable, annotation-free, and data-efficient 3D perception systems tailored for autonomous driving applications.

## Figures and Tables

**Table 1:** Comparison between the proposed LiGaussOcc and state-of-the-art methods on the 3D semantic occupancy prediction task using the nuScenes-OpenOccupancy validation set [11,44]. “GT” represents occupancy ground truth for supervision. “C” and “L” denote camera and LiDAR modalities, respectively. Since the “other flat” class is an invalid prompt for open-vocabulary models, this class was ignored during evaluation; ‘mIoU’ was calculated by ignoring the class.

Methods	Modality	GT	IoU	mIoU	■Barrier	■Bicycle	■Bus	■Car	■Const. Veh.	■Motorcycle	■Pedestrian	■Trafficcone	■Trailer	■Truck	■Drive. Surf.	■Sidewalk	■Terrain	■Manmade	■Vegetation
MonoScene [24]	C	✓	18.4	6.9	7.1	3.9	9.3	7.2	5.6	3.0	5.9	4.4	4.9	4.2	14.9	7.9	7.4	10.0	7.6
TPVFormer [25]	C	✓	15.3	7.7	9.3	4.1	11.3	10.1	5.2	4.3	5.9	5.3	6.8	6.5	13.6	8.3	8.0	9.2	8.2
C-CONet [11]	C	✓	20.1	12.4	13.2	8.1	15.4	17.2	6.3	11.2	10.0	8.3	4.7	12.1	31.4	18.7	16.3	4.8	8.2
JS3C-Net [29]	L	✓	30.2	12.3	14.2	3.4	13.6	12.0	7.2	4.3	7.3	6.8	9.2	9.1	27.9	14.9	16.2	14.0	24.9
LMSCNet [15]	L	✓	27.3	11.4	12.4	4.2	12.8	12.1	6.2	4.7	6.2	6.3	8.8	7.2	24.2	16.6	14.1	13.9	22.2
L-CONet [11]	L	✓	30.9	15.4	17.5	5.2	13.3	18.1	7.8	5.4	9.6	5.6	13.2	13.6	34.9	22.4	21.7	19.2	23.5
**LiGaussOcc (Ours)**	L	×	30.4	14.1	15.8	3.3	11.7	15.4	6.0	3.8	7.1	3.2	11.6	12.5	**35.4**	**22.6**	19.7	**19.6**	**23.8**

**Table 2:** Ablation study of main components.

EVI Module	AFF Module	IoU	mIoU
	✓	29.7	12.5
✓		30.1	13.2
✓	✓	**30.4**	**14.1**

**Table 3:** Ablation study of supervision methods.

Ground Truth	Volume Rendering	Splatting Rendering	IoU	mIoU
✓			31.1	15.8
	✓		30.2	13.7
		✓	30.4	14.1

**Table 4:** Ablation study of AFF.

Fusion Method	SC IoU	mIoU
Summation	30.2	13.6
Concatenation	29.9	13.1
AFF (ours)	**30.4**	**14.1**

## Data Availability

Two publicly available datasets were analyzed in this paper. The Nuscenes dataset can be found here: https://www.nuscenes.org/ (accessed on 1 July 2025). The OpenOccupancy dataset can be found at https://github.com/JeffWang987/OpenOccupancy (accessed on 1 July 2025).

## Abbreviations

The following abbreviations are used in this manuscript:

3D	three-dimensional
LiDAR	light detection and ranging
BEV	Bird’s-Eye View
2D	two-dimensional
CNN	convolutional neural network
IoU	Intersection over Union
mIou	mean IoU
FPN	Feature Pyramid Network
